# Smartphones and Learning: Evaluating the Focus of Recent Research

**DOI:** 10.3390/ejihpe13040056

**Published:** 2023-04-06

**Authors:** Kendall Hartley, Bobby Hoffman, Alberto Andújar

**Affiliations:** 1 Department of Teaching & Learning, University of Nevada, Las Vegas, NV 89154, USA; 2 College of Community Innovation and Education, University of Central Florida, Orlando, FL 32816, USA; 3 Faculty of Humanities, University of Almería, 04120 La Cañada, Spain

**Keywords:** smartphone, M-learning, smartphones and learning, bibliometrics

## Abstract

The smartphone has become integral to most aspects of students’ lives and is the primary conduit for accessing the internet. Objective research into the promise and dangers of this device is critical. While educational uses of the smartphone with young adults hold promise, the potential for harm is also present. While objectivity is valued, the focus of researchers can subjectively skew towards optimistic or pessimistic views of technology. The topics addressed in smartphone and learning research illuminate trends and potential biases in the field. This study investigates the issues addressed in smartphone and learning research in the past two years. These topics are compared with smartphone research in a similar field: psychology. The study, using a bibliometric approach, identified an overall negative arc of the literature towards topics such as addiction, depression, and anxiety in the psychology literature. The educational literature topics were comparatively more positive than psychology. Highly cited papers in both fields reflected explorations of adverse outcomes.

## 1. Introduction

The smartphone has become the primary conduit for school-aged youth to access the internet [1]. Objective research into the implications for smartphones and learning is of paramount importance. However, while objectivity is the goal, the topics, and research questions addressed can be skewed toward the positive or negative. Educational research into the use of smartphones has rightly focused on the impact on learning. With studies pointing to the adverse effects of excessive smartphone use [2] and findings indicating positive outcomes such as increased engagement [3,4], the appropriate guidance for educators is ambiguous. Investigations of smartphone use in non-education settings have also produced results that lack clear direction for practitioners. For example, introducing mental health professionals to the potential benefits of smartphone apps in reducing anxiety is encouraged [5]. Conversely, descriptions of negative consequences of excessive smartphone use (e.g., depression) are common [6,7].

For educational research to positively impact teaching and learning, the objective evaluation of the pros and cons of related innovations is essential. The topics that researchers choose to address can impact educational practice. For example, smartphone investigations addressing multitasking have noted mainly the adverse effects [8]. Studies focused on particular applications, such as augmented mobile virtual reality, emphasize affordances [9].

This work aims to identify the positive and negative impacts addressed in smartphone and learning-related research. While young adults are the primary participants in published smartphone research, this investigation will explore the comprehensive literature related to smartphones in two disciplines. This study explores the framing and potential research biases through a bibliometric analysis of the author keywords for recent smartphone and learning research. 

### 1.1. Smartphone and Learning Research Focus

By choosing topics and research questions, researchers can reveal biases regarding the role and influence of technology. The relationship between advocacy and objectivity is often problematic. For example, while many gambling researchers support government intervention, a lack of transparency regarding distinctions between advocacy and unbiased research can be evasive [10]. Scholars have criticized educational technology research for highlighting positive research outcomes that have a limited impact on classrooms [11]. New technologies often introduce capabilities that catch the attention of educators in search of improving teaching and learning. The enthusiasm for new technologies can introduce a confirmation bias when evaluating the utility of the innovation. The history of scientific research provides ample evidence that investigators are not immune to bias [12]. More recent work has illuminated the problems associated with perpetuating learning myths commonly associated with technologies [8]. Educators are inclined to accept misconceptions such as the digital native (i.e., the younger generations are more competent with technology) and the benefits of multitasking (i.e., you can accomplish more in less time when attending to multiple tasks simultaneously) when the face validity of these assertions is strong. 

As the latest and most widely adopted innovation introduced into classrooms, the smartphone has fostered tremendous multidisciplinary attention. Education is among those disciplines where the impact has been swift and extensive. Innovations with far-reaching educational implications, such as the desktop computer and the internet, progressed from classroom novelty to ubiquity in roughly twenty years (PC, ~1984–2004; Internet, ~1993–2013) [13,14]. The trajectory of the smartphone has been such that in about ten years (2009–2019), the device found its way into the hands of virtually every U.S. high school and university student [15]. 

While educators and researchers guided the introduction of computers and the internet to the classroom, smartphone presence in schools has been rapid and driven by learners of all ages. In a recent survey of 8–10 year-olds in rural Germany, researchers found that 67% of students owned a smartphone [16]. In the United States, 95% of 13–17 year-olds have access to a smartphone [1]. Educators and researchers now face a classroom reality that includes a pervasive and powerful technology component. How researchers investigate this innovation can have important implications for education. 

This study takes a broad view of the education literature, without constraints for settings or learners, to understand how researchers are studying smartphones. The context is then the smartphone research literature as represented in the Web of Science research database. 

The current research aims to explore the foci of smartphone and learning investigations. Specific objectives of the study include:

1. What are the topics framing smartphone and learning research?
2. Do the topics reflect a balanced view of the potential costs and benefits of smartphone use?

#### 1.1.1. Optimism

Advocates of using smartphones in teaching and learning highlight device capabilities, increased engagement, and the relationship to improved learning outcomes [17,18]. For instance, mobile devices can integrate innovative educational methods and develop high-level skills such as creativity, problem-solving, and communication [19]. Promising interventions are evident in a variety of educational disciplines. Using a smartphone app designed to promote self-regulated learning, researchers identified an increase in achievement-promoting behaviors among first-year university students compared to controls [20]. Mobile language learning has provided several examples of the potential of the smartphone to enhance learning. In a study of Slovak English language learning students, students viewed smartphone apps positively [21]. Others found using mobile instant messaging apps useful to assess students’ potential for language development [22]. In a recent meta-analysis of augmented reality studies, researchers found that the preponderance of recent investigations utilize mobile-based technologies [23]. The hope is that augmented reality can partially reduce cognitive load by eliminating the split-attention effect [24]. While certain types of augmented reality (e.g., spatial AR) minimize cognitive load, others, such as mobile vision-based AR, tend to increase extraneous cognitive load [23].

Beyond teaching and learning, smartphone interventions have demonstrated positive outcomes in physical fitness and mental well-being. Physical fitness smartphone applications are promising for weight loss and increased physical activity [25]. These apps use wearable devices for activity monitoring and social comparisons to improve the implementation fidelity of known efficacious interventions. Similarly, a meta-analysis of psychological interventions addressing anxiety with the smartphone found overall positive effects (g = 0.45) compared to controls [5]. The studies delivered a variety of interventions, such as cognitive bias modification, acceptance and commitment therapy, mindfulness training, breathing exercises, and self-awareness training. 

#### 1.1.2. Pessimism

One can contrast the promising research described above with similar research that often demonstrates less-than-ideal outcomes. 

#### Distraction

One of the smartphone’s key features is the complex system of notifications. Each installed app can interact with the phone’s OS to gain the user’s attention. The visual presence of a smartphone alone can distract the learner and reduce cognitive capacity [26]. The results of one study suggest that students may be distracted for over 200 h per year while attempting to study [27]. In an analysis of the relationship between planning modality (digital vs. non-digital) and class achievement, students opting for non-digital planning tools outperformed their digital-minded peers [28]. Digital distraction is not unique to the smartphone. The propensity to engage in off-task behavior is evident in numerous settings. A study of a one-to-one middle school computer initiative revealed that off-task behavior was more likely than on-task activity in the classroom [29]. 

#### Depression and Anxiety

The sudden mass adoption of the smartphone has engendered substantial concerns regarding mental health that are becoming more apparent as research catches up. In an analysis of the Korean Youth Health Behavior Survey, researchers found that individuals who were overdependent upon their smartphones were more than twice as likely to have a generalized anxiety disorder [30]. Accessing social media is by far the most common use of the smartphone. A recent analysis of social media use indicated that 53% of teens were on at least one social media platform ‘almost constantly’ [1]. In a six-month longitudinal comparison of 2500 young adults, those in the highest quartile of social media use were 2.7 times more likely to develop depressive symptoms than those in the lowest quartile [7]. 

#### Health

Smartphone use is also introducing increased physical fitness concerns. In examining the relationship between smartphone use and posture, researchers found that smartphone use introduces significant changes to the spine [31]. There is also a strong negative relationship between smartphone use and physical activity [32]. A study of Korean youth found a positive relationship between smartphone use and hedonic motives and a negative association with eudaimonic explanations [33].

In a recent study on the effect of mobile phone usage on sleep quality and academic performance, researchers indicated that mobile devices could disturb sleep rounds and consequently impact academic performance [34]. The adverse effects of sleep insufficiencies included absenteeism, trouble directing attention, and reduced motivation. A related study indicated that individuals who utilized information and communication technologies before bed were usually younger than thirty years old [35]. These students were also the group indicating the highest number of sleep disorders.

#### 1.1.3. Bibliometrics and Research Bias

This research aims to investigate the topics addressed in studies of smartphones and learning as well as the potential tendencies to investigate positive implications. In other words, are researchers favoring investigations that promote using smartphones for learning over studies that address concerns? Bibliometric analyses may aid an objective study of this question. 

Researchers can use various measures to ascertain the importance, interest, and distribution of reported research [36]. Trends in domain-specific research are reflected in indices such as the Social Science Citations Index and SCOPUS. Examples of trend analyses can be found in geosciences [37], artificial intelligence [38], and mobile learning [39]. Utilizing bibliometrics to investigate publication bias has occurred in a variety of settings. Inquiries into institutional bias in the *New England Journal* publications used bibliometric analysis to conclude that published articles did not favor Yale and Harvard authors [40,41]. An analysis of participant gender reporting in health sciences research revealed associations between the gender of the author(s) and the propensity to include sex-related data [42]. 

A recent bibliographic analysis of mobile learning revealed that smartphone research represents a substantial portion of the highly cited activity [39]. While the focus of the Goksu analysis was mobile learning, it reveals the rising importance of the smartphone for learning research. The substantive distinctions between smartphone and mobile learning research necessitate a different review [43]. A content and bibliometric analysis of augmented reality (AR) and science education research revealed that the preponderance of studies utilized smartphones [44]. This work will use the term smartphone to better focus on the relevant literature under review. In educational research, the smartphone is often subsumed into mobile learning despite its unique and comprehensive role in society. One example of this expansive role is the rising concern over mental health implications [6,7,45]. 

## 2. Methods

To address the question of the balanced treatment of the smartphone in learning research, a collection of recent research articles was generated using the author keyword *smartphone*. From this core collection of articles, two datasets were generated based on the Web of Science categories applied to the research articles. In general terms (with specifics to follow), the primary dataset included research that was education-focused. A comparison dataset was required to determine if the education-focused literature is neutral in the treatment of smartphones and learning. For this study, psychology-focused categories provided the most reasonable choice for comparison. Several attributes of the psychology literature supported it as an appropriate choice. For example, as noted earlier, psychology has seen clear evidence of positive [5] and negative [7] consequences of smartphone use. In addition, the psychology categories include a substantial number of studies that address ICT concerns and utilize smartphones as a keyword. Education and psychology as social sciences are comparable in methods, norms, and applications.

The two article datasets were developed utilizing the Web of Science search and export functions [46]. Web of Science is a multidisciplinary platform designed to facilitate the search and collection of research literature. The platform can be used to search for research articles based on a wide variety of parameters. The parameters of interest for this work are the Web of Science categories, publication year, and author keywords. 

The first education dataset was created to represent recent educational-focused research that used the word smartphone as an author keyword. This education dataset was generated by searching the SSCI (via the Web of Science) for research articles that were categorized as in general education (see the appendix for detailed search parameters). The second psychology dataset was generated by searching the SSCI for research articles that were categorized as psychology. 

A series of keyword analyses were conducted with recent smartphone-related studies to address the research questions. The manuscripts reviewed were cataloged in the Social Sciences Citation Index (SSCI). The analyses were completed with R software [47], RStudio IDE [48] and the Bibliometrix package [49].

One approach to determining the general trends in research is to review the keywords associated with the topic of interest. Sentiment analysis lexicons can provide a general classification as positive (e.g., satisfaction, skills, care) or negative (e.g., depression, anxiety, addiction). The top twenty author-chosen keywords and sentiments (based on MPQA Subjectivity Lexicon [50,51]) for each dataset were compared (see Table 1).

**Figure 1:**
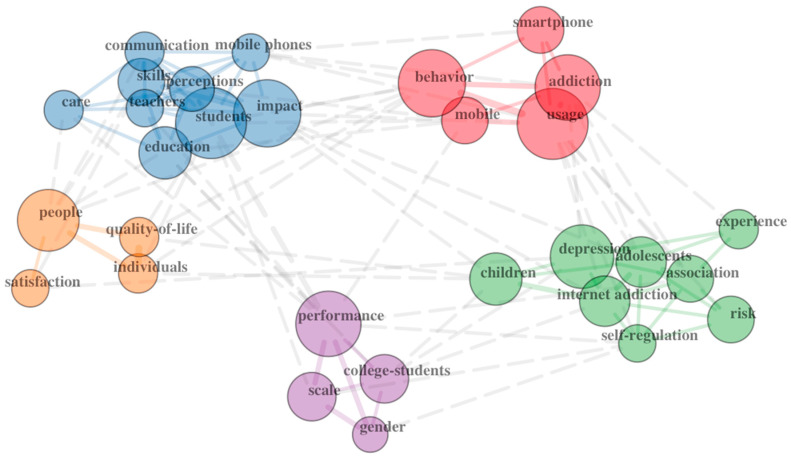
Education author keyword co-associations.

**Figure 2:**
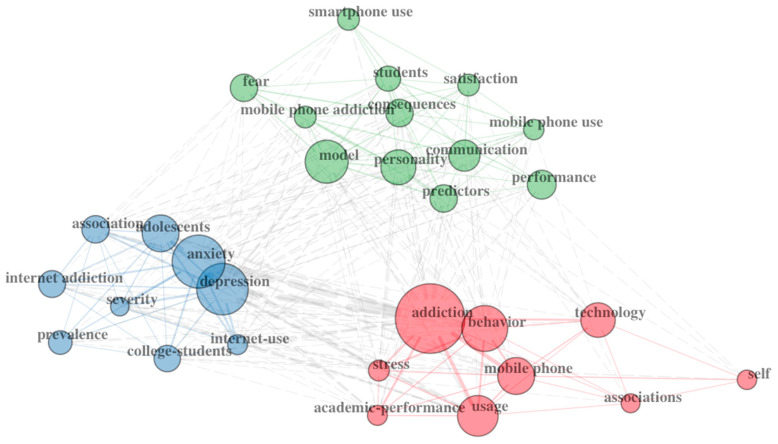
Psychology author keyword co-associations.

A network map of the co-occurrence of the keywords can also prove helpful in determining how topics cluster within research studies and between domains. See Figure 1 (Education) and Figure 2 (Psychology) for examples. 

The works cited in each of these datasets can communicate the type of work valued by each discipline. Table 2 presents a tabulation of the top papers cited by the papers in each dataset and highlights those that are receiving the greatest attention *within* the respective disciplines.

## 3. Results

### 3.1. Keyword Frequency

The keyword comparisons between the education and psychology datasets (Table 1) reveal a distinct difference in emphases. Sentiment analysis lexicons can be used to guide descriptions of these emphases. For this discussion, keywords are classified as positive (e.g., satisfaction, skills, care) and negative (e.g., depression, anxiety, addiction) based on the MPQA Subjectivity Lexicon [51,60]. The keywords chosen by the authors for the psychology document overwhelmingly favor the negative consequences of smartphone use. The education keywords also reveal problematic topics but to a far lesser degree.

### 3.2. Cluster Maps 

The co-association cluster maps (Figure 1 and Figure 2) also reveal distinct differences. The psychology map includes three clusters, two of which reflect largely negative topics. The education cluster map included five clusters. Three of the clusters include terms reflecting a positive view of smartphones. One cluster is mainly positive but includes the term addiction. The fifth cluster reflects a negative view of smartphones. A holistic appraisal of the two maps supports a positivity bias in education research compared to psychology research.

### 3.3. Top Cited Articles

A contrasting picture emerges when reviewing the top-cited articles within each dataset. As noted above, this comparison can provide an indication of the works that are valued within the respective disciplines. The potential negative consequences of smartphone use appear prominently in each list. Each of the top-cited psychology papers addresses smartphone addiction. One article in each list describes a well-accepted measure of smartphone addiction, the smartphone addiction scale. While the education articles are all negative, one highly cited paper focuses on balancing the positive and negative implications of the smartphone for learning. 

## 4. Discussion

This study aimed to describe the topics addressed in smartphone and learning research. In addition, the investigation aimed to gauge the balance of the research conducted over the past two years with an eye toward potential positivity bias. The findings revealed a strong interest in addiction, anxiety, and depression in the psychology literature reviewed. This is consistent with recent commentaries regarding psychological research [61]. While beneficial uses of the smartphone are present in this literature [62], they are far less prevalent in number and cited less frequently. In comparison, the education literature reviewed covers more positive [63] and neutral topics. The education literature is weighted toward topics that highlight the potential benefits of the smartphone for teaching and learning. Cautionary studies are available in the education literature, and those seem to receive an outsized amount of attention as measured by citations. 

### 4.1. Implications and Significance

It is incumbent upon education researchers to pursue inquiries as objectively as possible. While it is tempting to envision clear benefits from the latest and most available technologies, the history of educational technology research is rife with examples of investments that ultimately provide limited improvement to learning outcomes [11]. 

Investigations should provide sound theoretical support, including a description of how the smartphone can improve learning outcomes. This support should provide a risk and reward analysis that incorporates potential threats to learning, such as distraction. As indicated by the psychology research literature, there are substantive concerns surrounding the excessive use of smartphones. These concerns are particularly relevant to younger learners [64]. It is also evident that the distractions presented by the smartphone encourage off-task behaviors [8].

This is not to say that smartphones should be banned from learning environments. Students with a healthy and moderated connection with the smartphone can certainly benefit from the myriad of capabilities that can be brought to bear towards learning. What a healthy and moderated connection looks like is worthy of future research. For example, how well do students understand the management and consequences of smartphone notifications? Do students understand how much use is too much or *problematic*?

Practitioners and researchers are not immune to the allure of the latest technologies. However, they are also more likely to understand the risk and rewards associated with using smartphones in the classroom. The rapid ascent of the smartphone into the backpack of nearly every young adult has been immensely consequential for educators battling for their attention. This work further supports the need to critically analyze the questions being addressed by educational researchers as they relate to the latest technologies.

### 4.2. Limitations and Further Research

There are several notable limitations to the current investigation. The studies reviewed represent a recent but small sample of the literature produced since the broad adoption of the smartphone. This choice was predicated on the time lag between adoption and the publication of the research. It is also evident that the rapid adoption of the smartphone has resulted in a fast-changing learning context. 

The choice of disciplines for comparison could be challenged on the grounds that the type of research is fundamentally different, and distinctions are representative of those differences alone. For example, educational researchers are generally charged with looking for ways to improve the learning environment. Psychological researchers may trend toward concerns regarding obstacles to well-being. These tendencies may be independent of technology use, and a broader review of the literature might reveal similar biases.

Another limitation stems from the use of author-chosen keywords and citations. Using keywords is a valid but imperfect measure of the direction of the research. Author-chosen keywords may not completely capture the nature of the work. However, it could also be argued that this is a strength in that the nature of the inquiry is to ascertain the researcher’s bias through their own characterization of the work. Citations are also a useful but imperfect measure of the attention given to a research study. A more sophisticated sentiment analysis or manual content analysis of complete articles could produce a more nuanced description of the observed research. 

Future research could extend existing work that has reviewed mobile learning authors, journals, and countries (e.g., [39]) to better understand the variations in topics addressed. In particular, an analysis focused on educational technology journals provides additional insights into the aims of this study regarding biases of researchers with a focus on technology. 

In more general terms, future smartphone and learning research should reflect a more balanced treatment of the educational implications of introducing this powerful technology into the classroom. There is a robust literature available on the appropriate use of multimedia technologies in support of learning [65]. Research guidance regarding the misuse of multimedia also predates the mainstream adoption of the smartphone [65]. This literature can provide theoretical support for innovative learning approaches that account for potential adverse impacts. It is not necessary to reinvent the wheel each time a new technology is introduced.

## Figures and Tables

**Table 1:** Top twenty author-chosen keywords in datasets 2 (education) and 3 (psychology).

	Psychology			Education		
	Author Keywords	Sentiment ^1^	Articles	Author Keywords	Sentiment ^1^	Articles
1	smartphone		32	smartphone		47
2	smartphone *addiction*	Negative	31	mobile *learning*	Positive	10
3	*problematic* smartphone use	Negative	27	smartphone *addiction*	Negative	6
4	smartphone use		19	addiction	Negative	4
5	adolescents	Neutral	10	education		4
6	depression	Negative	10	smartphone usage		4
7	*fear* of missing out	Negative	10	university students		4
8	anxiety	Negative	9	disability		3
9	nomo*phobia*	Negative	6	higher education		3
10	phubbing (ignore)	Negative	6	intellectual		3
11	internet *addiction*	Negative	5	smartphone app		3
12	attachment		4	smartphone applications		3
13	distraction	Negative	4	smartphone use		3
14	mobile		4	tablet		3
15	smartphone dependency		4	technology		3
16	social media		4	adolescents	Neutral	2
17	addiction	Negative	3	communication		2
18	adolescence	Neutral	3	electronic media		2
19	college students		3	gender		2
20	loneliness	Negative	3	high school		2

^1^ MPQA Subjectivity Lexicon polarity. Italicized words in phrases were found in the lexicon. Blank entries are not in the lexicon.

**Table 2:** Five most-cited papers from articles using the keyword smartphone.

First Author	Year	Journal Abbreviation	Title	Reference
*Education*				
Brown, I.	2013	*Intellect. Dev. Disabil.*	Quality of life indicators for individuals with intellectual disabilities: Extending current practice	[52]
Cha, S.-S.	2018	*Health Psychol. Open*	Smartphone use and smartphone addiction in middle school students in Korea: Prevalence, social networking service, and game use	[53]
Kwon, M.	2013	*PLoS ONE*	Development and validation of a smartphone addiction scale (SAS)	[54]
Van Deursen, A.J.	2015	*Comput. Hum. Behav.*	Modeling habitual and addictive smartphone behavior: The role of smartphone usage types, emotional intelligence, social stress, self-regulation, age, and gender	[55]
Anshari, M.	2017	*Educ. Inf. Technol.*	Smartphones usage in the classrooms: Learning aid or interference?	[17]
*Psychology*				
Elhai, J.D.	2017	*J. Affect. Disord.*	Problematic smartphone use: A conceptual overview and systematic review of relations with anxiety and depression psychopathology	[56]
Billieux, J.	2015	*Curr. Addict. Rep.*	Can disordered mobile phone use be considered a behavioral addiction? An update on current evidence and a comprehensive model for future research	[57]
Kwon, M.	2013	*PLoS ONE*	The smartphone addiction scale: development and validation of a short version for adolescents	[58]
Van Deursen, A.J.	2015	*Comput. Hum. Behav.*	Modeling habitual and addictive smartphone behavior: The role of smartphone usage types, emotional intelligence, social stress, self-regulation, age, and gender	[55]
Kardefelt-Winther, D.	2014	*Comput. Hum. Behav.*	A conceptual and methodological critique of internet addiction research: Towards a model of compensatory internet use	[59]

## Data Availability

Source data are available via the links in the Appendix A.

## Appendix A

*Core Dataset*

https://www.webofscience.com/wos/woscc/summary/c9a4e2d8-67e4-40f7-8b91-8c2010b94c60-0ed03a93/relevance/1 (Access date: 6 February 2022)

Editions: SSCI and ESCI

Publication year: 2020–2021

Author keywords [AK]: smartphone (anywhere in keywords, e.g., “problematic smartphone use” is included)

Document type: Articles

1386 results

Excluded non-behavioral WOS Categories (e.g., chemistry, physics, general medicine, surgery, environmental sciences. Etc.)

973 results

*Dataset #1—Education*

https://www.webofscience.com/wos/woscc/summary/f3b33719-001b-4588-8f75-e317b4b424f7-0ed06b5a/relevance/1 (Access date: 6 February 2022)

*Dataset #1 refined with:*

WOS Education Categories (74)

Education Educational Research (61)

Education Scientific Disciplines (12)

Education Special (7)

*Dataset #2—Psychology*

https://www.webofscience.com/wos/woscc/summary/f0fae222-c098-44c4-8a19-b975a11a63f0-0ed078f3/relevance/1 (Access date: 6 February 2022)

*Dataset #1 refined with:*

WOS Education Categories (116)

Psychology Multidisciplinary (103)

Psychology Experimental (40)
